# Ischaemic mitral regurgitation in coronary revascularization: A critical gap in surgical guidelines

**DOI:** 10.34172/jcvtr.025.33085

**Published:** 2025-06-28

**Authors:** Rahul Bhushan, Vijay Grover

**Affiliations:** Department of CTVS, ABVIMS and Dr RML Hospital, New Delhi, 110001, India

**Keywords:** Ischaemic mitral regurgitation, Coronary artery disease, Coronary artery bypass grafting

## Abstract

Ischemic mitral regurgitation (IMR) presents a clinical challenge amidst evolving treatment paradigms, particularly when accompanied by coronary artery disease (CAD). Controversies persist regarding the optimal surgical approach, resulting in a lack of definitive guidelines. A comprehensive review of seminal studies from 2000 to 2020 was conducted to elucidate the evolving discourse and treatment landscape for IMR. Studies encompassing varied interventions, including coronary revascularization and concomitant mitral valve procedures, were scrutinized to gauge their impact on patient outcomes. Early studies diverged in advocating for or against adjunct mitral valve intervention during coronary artery bypass grafting (CABG) in IMR patients. Subsequent trials like the POINT and RIME trials highlighted benefits associated with concomitant mitral interventions. However, the Cardiothoracic Surgical Trials Network (CTSN) trials raised concerns regarding adverse events and recurrence rates post-mitral repair.The ambiguity in guidelines for IMR management persists, leaving surgeons to navigate individualized treatment decisions. Recommendations from the American Heart Association (AHA) offer moderate support for mitral valve interventions, yet a clear consensus remains elusive. The necessity for refined guidelines reflecting current evidence is imperative to optimize outcomes in IMR patients.

## Introduction

 Chronic ischemic mitral regurgitation has remained a contentious subject for over two decades, persisting amidst unresolved debates, ambiguous recommendations, limited evidence-based outcomes, and divergent surgical approaches in cases involving concurrent coronary revascularization and mitral valve procedures within a compromised cardiac framework. Notably, the operative risks associated with ischemic mitral regurgitation far exceed the cumulative risks expected from either isolated mitral valve pathology or ischemic heart conditions.^1^

 The absence of clear directives or a standardized approach perplexes surgeons faced with coronary artery disease (CAD) coupled with ischemic mitral regurgitation (MR). This dilemma is further compounded by the frequent coexistence of left ventricular (LV) dysfunction, presenting a formidable challenge when contemplating valvular interventions in the setting of a compromised cardiac function.^2^ Complications intensify in regions like the Indian subcontinent, where a pronounced inclination towards off-pump coronary revascularization prevails due to financial constraints and numerous other limitations, adding complexity to an already intricate scenario.

 Our study endeavours to decipher the most optimal evidence-based recommendations and confront the intricate challenge posed by ischemic MR, seeking an adaptable and practical approach amid these complexities.

## Methods

###  Search Strategy 

 The systematic review was conducted using PubMed, EMBASE, and Cochrane Library databases. Articles from December 31, 2000, to December 31, 2022, were included, with exclusion criteria applied to publications before 2001. Searches were limited to English-language articles. Pilot searches helped determine relevant Medical Subject Headings (MeSH) terms, including “cardiac surgical procedures,” “Ischaemic mitral regurgitation,” “mitral valve/therapy,” “coronary artery bypass,” and “Health-related Quality of life.” Boolean logic operators (“AND” and “OR”) were used to refine the search algorithm.

###  Eligibility Criteria 

 The inclusion criteria comprised randomized controlled trials (RCTs), cohort studies, and cross-sectional studies assessing Quality of Life (QoL) in patients with ischaemic mitral regurgitation undergoing coronary revascularization. Exclusion criteria included narrative reviews, abstracts, case reports, and editorials.

###  Data Collection and Extraction

- *Selection of Studies:* Two authors independently screened article titles and abstracts based on inclusion/exclusion criteria. Discrepancies were resolved through discussion with a third author. Full-text manuscripts of relevant articles were evaluated, and additional relevant studies were identified through a hand check of reference lists.
- *Assessment of Study Quality:* The quality assessment followed the National Institutes of Health’s tool for controlled intervention, observational cohort, and cross-sectional studies. Criteria were sorted into primary, secondary, and tertiary levels based on their importance for study quality. Details of the sorting methodology are provided in the addendum. Ratings of “good,” “fair,” or “poor” were assigned based on meeting primary and secondary criteria.

###  Quality Assessment 

 Cross-sectional studies, while potentially rated as “good,” were capped at “fair” due to their inherently less robust design. Quality assessment was independently conducted by two authors, with discrepancies resolved through discussion with a third author.

## Results

###  Evolution of Debate

 The turn of the 21st century marked the onset of debates among patients undergoing coronary revascularization with concurrent coronary artery disease (CAD) and mitral regurgitation (MR). Landmark studies, presented chronologically, track the progression of this debate and the emerging consensus among surgeons regarding effective approaches to address this combined entity.

###  Intervention Vs No Intervention: The Emergence of Controversy

 The genesis of controversy surfaced with pioneering studies investigating coronary artery bypass grafting (CABG) alone versus its combination with mitral valve intervention in moderate ischemic mitral regurgitation (IMR).

 In 2001, Aklog et al conducted a seminal study published in Circulation, evaluating the efficacy of CABG as a standalone therapy for moderate IMR. Spanning seven years and encompassing 136 patients, their findings contested the notion of CABG alone as the optimal therapeutic avenue. Instead, they advocated for concomitant mitral valve intervention to achieve superior outcomes.^3^

 However, diverging views emerged swiftly in 2002 through the work of Tolis et al. Their extensive 10-year retrospective study involving 183 patients undergoing CABG with MR unveiled contrasting outcomes. The study suggested that isolated CABG sufficed for patients with moderate ischemic MR, showcasing notable long-term enhancements in left ventricular function and MR severity based on LV remodelling. Consequently, this study discouraged the need for concomitant mitral intervention alongside CABG.^4^

 Adding to this discourse, a seven-year retrospective study by Reece et al in 2004 furthered the dialogue. This study, involving 110 patients randomized into groups undergoing either mitral valve repair or replacement alongside CABG for ischemic severe MR, offered distinct insights. Contrary to earlier beliefs, it revealed that mitral repair exhibited superiority over replacement in patients undergoing coronary revascularization concurrently with mitral intervention.^5^ This nuanced understanding propelled the once-neglected entity into the spotlight, triggering open discussions and debates among surgeons regarding the optimal approach for this subgroup of patients.

###  Platelet-Oriented Inhibition in New TIA and Minor Ischemic Stroke (Point Trial): Unveiling the Significance of Concomitant Mitral Valve Repair

 The pivotal Point Trial, published in 2009, marked the inception of prospective randomized controlled trials comparing the efficacy of isolated CABG versus CABG combined with Mitral Valve Repair (MV Repair) in patients with moderate MR. This single-centre Italian study conducted over a four-year duration enrolled 102 patients. Among them, 48 patients underwent CABG coupled with restrictive mitral valve annuloplasty (MVA), while 54 underwent solely CABG procedures.

 The trial’s paramount findings unveiled compelling outcomes. The subgroup undergoing concomitant mitral annuloplasty during coronary revascularization exhibited noteworthy advantages. Patients experienced significantly reduced symptoms and reported a better quality of life compared to those who underwent isolated CABG. Furthermore, the post-operative echocardiological assessments revealed pivotal differences. Individuals who underwent isolated CABG showcased poorer left ventricular function and witnessed the progression of residual MR, underscoring the added value of concomitant mitral intervention.^6^

 The implications of these findings reverberated across clinical practice, emphasizing the potential benefits of simultaneous mitral valve repair during coronary revascularization, not only in symptom alleviation but also in mitigating adverse post-operative cardiac outcomes.

###  Randomized Ischemic Mitral Evaluation (RIME Trial): Enhancing Cardiac Function with Concomitant Mitral Valve Repair

 The 2012 Randomized Ischemic Mitral Evaluation (RIME) trial, led by Chan et al^7^ aimed to assess the benefits of adding Mitral Valve (MV) Repair to Coronary Artery Bypass Grafting (CABG) in moderate Ischemic Mitral Regurgitation (IMR). Seventy-three patients undergoing coronary revascularization were randomized into groups receiving either CABG alone or combined with mitral intervention.

 The findings favoured the concomitant mitral annuloplasty group. This cohort displayed improved functional capacity, reduced MR severity, significant left ventricular reverse remodelling, and lower B-type natriuretic peptide (BNP) levels compared to those undergoing solely CABG procedures.^7^ These results highlighted the potential of combining mitral valve repair with CABG in enhancing cardiac function and mitigating MR severity in moderate IMR cases.

###  Shift in Severe MR Diagnosis

 Between 2014 and 2017, the American Heart Association (AHA) introduced revised guidelines for diagnosing severe Mitral Regurgitation (MR), elevating Echocardiogram (ECHO) thresholds. This adjustment aimed to potentially mitigate excessive interventions linked to concomitant mitral procedures, although it was supported by limited evidence.^8^

###  The Game changer: CTSN Trial- Paving way for current recommendations

 The largest prospective randomized trial to date on IMR was conducted by the Cardiothoracic Surgical Trials Network (CTSN). The first arm of CTSN multicenter trial involved 22 institutions with 251 patients with severe ischemic MR who were randomized into either mitral repair vs replacement group and were followed for 2 years. Although no significant difference was found in reverse remodeling amongst both groups, the mitral repair group had more frequent adverse events as well as higher recurrence of regurgitation in comparison to replacement group.^9^

 Similarly, the other arm of CTSN trial studying group of moderate mitral regurgitation with coronary artery disease published its finding same year. The trial comprised of 301 patients randomized to CABG alone vs combine CABG with mitral intervention group. The study concluded that additional mitral valve repair with CABG in subgroup with moderate MR did not yield added advantage in terms of left ventricular reverse remodeling. In addition, more adverse events were noted in the same subgroup undergoing concomitant procedure.^10,11^

###  2017 AHA Update: Refined Guidance for Severe Ischemic MR

 The American Heart Association (AHA) incorporated insights from the CTSN Trial into their 2017 guidelines for severe Ischemic Mitral Regurgitation (IMR) management:

- *MV Replacement:* Recommended for symptomatic patients (stage D) despite guideline-directed therapies, presenting basal aneurysm/dyskinesis, significant leaflet tethering, or severe left ventricular dilation (Class 2a, Level of Evidence B).
- *MV Repair:* Suggested for symptomatic patients (stage D) lacking specific indicators, involving an undersized complete rigid annuloplasty ring (Class 2b, Level of Evidence B).

These nuanced guidelines aimed to individualize treatment decisions, tailoring MV replacement or repair based on symptom severity and associated cardiac conditions, shaping refined strategies for severe Ischemic MR.

###  2020 AHA guidelines for management of valvular heart disease

 The last updated recommendation came out from AHA in 2020 which briefly discusses on entity of ischaemic MR only adding fuel to fire.^12^

###  2020 AHA Valvular Heart Disease Guidelines

 The 2020 AHA update briefly addresses Ischaemic Mitral Regurgitation (MR), focusing on:

- *Moderate Ischemic MR:* Regrettably, the 2020 recommendations omit direct guidance on moderate secondary MR. Thus, drawing inference from the 2017 AHA and American Association of Thoracic surgeons (AATS) recommendations, a Class 2B suggestion is proposed for adding mitral valve repair to coronary revascularization in the moderate MR subgroup.
- *Severe Ischaemic MR:* The guideline maintains a Level 2A recommendation, advocating concurrent mitral intervention during coronary revascularization for severe MR, referencing insights from the Surgical Treatment for Ischemic heart failure (STICH) Trial. Notably, the recommendation for mitral valve replacement is subtly downgraded from Class 2A to Class 2B compared to the 2017 update by AHA.

However, very deftly, the recommendation to perform a mitral valve replacement was downgraded from class 2A to class 2B in comparison to 2017 update by AHA. (Table 1)

**Table 1:** Major studies on Ischaemic mitral regurgitation

**Studies**	**Year, Journal**	**Participants**	**Inference**
Aklog et al^3^	2001, Circulation	136	Need for concomitant mitral intervention alongside CABG
Tolis et al^4^	2002, Ann Thorac Surg	183	Adequacy of isolated CABG for moderate IMR
Reece et al^5^	2009, J Thorac Cardiovasc Surg	110	Favourable outcomes with mitral repair over replacement
POINT Trial^6^	2009, J Thorac Cardiovasc Surg	102	Improved outcomes with concomitant mitral annuloplasty
RIME Trial^7^	2012, Circulation	73	Favourable outcomes with concomitant mitral annuloplasty
CTSN Trial^8^	2016, NEJM	251	Higher adverse events and regurgitation recurrence in mitral repair group
CTSN Trial 2^9^	2016, NEJM	301	No added advantage in LV reverse remodelling with additional mitral valve repair in moderate MR

 Meanwhile, findings from Endovascular Valve Edge-to-Edge Repair Study (EVEREST) trial were incorporated in American College of Cardiology/American Heart Association (ACC/AHA) guidelines of valvular heart disease considering transcatheter edge-to-edge mitral valve TEER as a reasonable option in symptomatic patients with chronic primary or secondary severe MR with high or prohibitive surgical risk and favourable anatomical features.^13^

 According to the 2020 AHA recommendations, guideline-directed medical therapy is advised for patients with chronic severe secondary mitral regurgitation who are not undergoing revascularization or mitral valve surgery. This is a Class I recommendation. Additionally, a Class IIa recommendation for TEER in these patients is provided in the recent guidelines.

 However, latest European Society of Cardiology (ESC) recommendations states class 1 recommendation for concomitant valvular surgery in severe symptomatic secondary mitral regurgitation while giving a Class IIa recommendation for TEER just like their counterparts. The recommendations once again leaves the area of opacity as no mention of moderate MR is made nor a discussion of repair versus replacement is made in surgical approach.^14^

## Discussion

 Functional ischemic mitral regurgitation commonly accompanies coronary artery disease (CAD). The absence of clear evidence-backed guidelines leaves a void in determining the optimal surgical approach for this patient subgroup (Table 1). As the ventricle enlarges, the mitral annulus widens and leaflet tethering occurs due to papillary muscle displacement, leading to functional mitral regurgitation. Leaflet tethering refers to the restriction of the mitral valve leaflets’ movement, a phenomenon often seen in conditions like ischaemic mitral regurgitation (IMR). Post-revascularization, the belief was that left ventricular (LV) remodelling would gradually reduce the severity of MR.^8^ However, this notion was challenged, particularly in severe cases exhibiting heart failure features where significant remodelling was not achievable. In such cases, a concurrent valvular intervention became imperative for surgical success.^15,16,17^

 An extensive review study by CG Mihos et al suggested that additional subvalvular repair, alongside mitral annuloplasty, should be a routine to enhance LV remodelling and improve outcomes.^18^ Similarly, F Nappi et al’s RCT found that including papillary muscle approximation with mitral annuloplasty during CABG with concurrent mitral intervention led to better long-term surgical outcomes.^18,19^

 Standard recommendations lack clear guidelines regarding intervention in ischemic mitral regurgitation in subset of moderate MR. In cases of moderate ischemic MR, a weak recommendation of 2B was given for a concurrent mitral valve surgical repair in 2017 guidelines.^18^ The latest 2020 update on valvular heart disease does not even address moderate ischemic MR, leaving surgeons to adopt a patient-tailored approach. Surgeons must evaluate whether concomitant valvular intervention could benefit patients without increasing morbidity/mortality risk. Variable surgical outcomes based on surgeon experience and the ideal repair strategy further complicate the preferred approach in such cases.^20^

 Even in severe ischemic MR cases, while the latest AHA 2020 update offers a level 2A recommendation for addressing the mitral valve during coronary revascularization, ambiguity persists regarding valve repair or replacement. The recommendation for valve replacement over repair has been downgraded from 2A to level 2B. The 2020 downgrade reflects a growing body of evidence suggesting that mitral valve repair is often more favourable than replacement in patients with ischaemic mitral regurgitation, particularly in terms of survival rates and preservation of ventricular function. Clinical trials and observational studies found that valve repair tends to be associated with lower mortality rates, fewer complications, and better quality of life outcomes compared to replacement in certain patient groups. Also, Mitral valve replacement, especially without chordal-sparing techniques, can further impair the left ventricular function by disrupting the normal tethering effect of the mitral apparatus.

 Surgeons must rely on their discretion due to the lack of clear guidelines. The inclusion of transcatheter intervention as a level 2A recommendation further clouds the choice between surgical or transcatheter approaches.^20,21^

 Further studies are needed for comparing surgical mitral valve repair/replacement to newer transcatheter mitral valve repair options, such as MitraClip, over long-term follow-up periods are limited. Also, Studies that explore the benefits of early intervention in moderate cases vs. conservative management could offer insights into whether earlier repair or transcatheter intervention might prevent disease progression or heart failure. Finally, more studies are needed to identify patient characteristics that predict better outcomes with repair rather than replacement.

## Conclusion

 The management of ischemic mitral regurgitation alongside coronary artery disease poses a clinical challenge due to the absence of clear-cut guidelines. Evolving perceptions about left ventricular remodelling and the role of concomitant valvular interventions have reshaped the discourse within surgical approaches. While some studies advocate for additional subvalvular repair to augment left ventricular remodelling and enhance outcomes, a lack of consensus among standard authorities leads to ambiguity in surgical decision-making for both moderate and severe ischemic MR cases.

 The latest guidelines provide only faint suggestions, leaving surgeons to rely on individual judgment and experience. Surgeons must weigh the risks and benefits of concomitant valvular interventions, considering the patient’s clinical status and surgical expertise. The ongoing debate between valve repair and replacement further complicates the decision-making process.

 Moving forward, comprehensive studies and collaborative efforts are imperative to formulate evidence-based guidelines that can streamline surgical strategies and ensure optimal outcomes for patients grappling with ischemic mitral regurgitation alongside coronary artery disease.

## Competing Interests

 The authors have no conflicts of interest to disclose.
